# Association between Catechol-O-Methyltransferase Val158Met (158G/A) Polymorphism and Suicide Susceptibility: A Meta-analysis

**Published:** 2017-06-24

**Authors:** Tahereh Sadeghiyeh, Fatemeh Hosseini Biouki, Mahta Mazaheri, Masoud ZareShehneh, Hossein Neamatzadeh, Zahra Poursharif

**Affiliations:** ^1^ Department of Psychology, School of Medicine, Shahid Sadoughi University of Medical Sciences, Yazd, Iran; ^2^ Department of Medical Genetics, School of Medicine, Shahid Sadoughi University of Medical Sciences, Yazd, Iran; ^3^ Mother and Newborn Health Research Center, School of Medicine, Shahid Sadoughi University of Medical Sciences, Yazd, Iran

**Keywords:** Suicide, Catechol-O-methyltransferase, Genetic association, Meta-analysis, Susceptibility

## Abstract

**Background:** Common functional Val158Met polymorphism in the Catechol-O-methyltransferase (COMT) gene may have an impact on an individual’s susceptibility to suicide, but individually published results are inconclusive. Therefore, we performed this meta-analysis to provide a more precise estimation of the association between COMT 158G/A (COMT Val158Met) polymorphism and suicide susceptibility.

**
Study design: ** A cross-sectional study.

**Methods:** This systematic review and meta-analysis is a comprehensive literature search of PubMed, Scopus, Web of Science and Google Scholar databases was conducted on case-control studies published up to Mar 2017. Crude odds ratios (ORs) with 95% confidence intervals (CIs) were calculated.

**Results:** We identified 14 eligible case-control studies, including 2353 suicide attempters and 2593 controls. The pooled results indicated that COMT 158G/A (COMT Val158Met) polymorphism was not significantly associated with increased overall suicide risk. The same results were revealed based on ethnicity, Hardy–Weinberg equilibrium (HWE) status and genotyping technique. However, there was significant association between COMT Val158Met polymorphism and suicide risk among females under the homozygote (AA vs. GG: OR=1.829, 95% CI=1.158-2.889, *P*=0.010) and recessive (AA vs. AG +GG: OR = 1.787, 95% CI=1.195, 2.671, *P*=0.005) models, but not among males.

**Conclusions:** COMT 158G/A (COMT Val158Met) polymorphism was associated with suicide susceptibility only in females.

## Introduction

Suicidal behavioris one of the main public health problems worldwide, which include a wide spectrum of self-destructive actions^1^. Approximately, one million people die by suicide each year worldwide. With a prevalence rate of 0.0145% and suicide accounting for 1.5% of death by all causes, it is the 10th leading cause of mortality worldwide^2^. The etiology of suicide due to a combined effect of genetic and external factors is not known exactly^3^. Social factors alone cannot fully explain suicidal behavior. However, some studies have indicated a strong genetic component to susceptibility to suicidal behavior^4^.

Catechol-O-methyltransferase (COMT) is an enzyme metabolizing noradrenaline in the synaptic cleft^5^. Association studies of dopamine receptor genes and COMT in schizophrenia and/or ADHD subjects report genetic associations with various domains of cognitive function, including executive function, verbal and working memory, attention, and performance monitoring. Therefore, it was suggested that COMT could also play a role in the susceptibility to suicidal behavior^6, 7^. The COMT gene has a common functional polymorphism, val158met (rs4680) variant^8, 9^. This polymorphism is due to a G to A transition at codon 158 of the membrane-bound form of COMT, which corresponds to codon 108 of the soluble form of COMT, resulting in a valine (Val) to methionine (Met) substitution^9^.

The genetic component of suicidal behaviorwas originally founded by family, adoption and twin studies. Its heritability is in the range between 30%–55%^10, 11^. A family history of suicide is a strong risk factor for suicide and suicide attempt^11^. The COMT gene is located on the long arm of chromosome 22 at 22q11; spans 28 kb and contains six exons^9^. The function of COMT polymorphism in several psychotic conditions, such as schizophrenia, bipolar disorder, major depressive disorder, obsessive-compulsive disorder, and Parkinson’s disease, and the putative effects of COMT on suicide have been proposed^12, 13^.

Several case-control studies have reported the association between COMT polymorphism and suicide susceptibility^8, 9, 18, 29^, but the results are inconclusive partially because of the possible small effect of the polymorphism on suicide risk and the small sample in some published study. Therefore, we performed a meta-analysis to derive a precise estimate of this association.

## Methods

### Publication Search

We searched the electronic PubMed, Scopus, Web of Knowledge, and Google scholar database to identify all case–control studies examined the association between COMT Val158Met (158G/A) polymorphism and suicide risk up to Mar 2017. The keywords were as follows: ‘’Catechol-O-methyltransferase’’, ‘’COMT gene’’, ‘’COMT Val158Met’’, ‘’COMT 158G/A’’, ‘’rs4680’’, ‘‘polymorphism’’, ‘‘genotype’’, ‘‘variant’’, ‘’mutation’’, suicide’’, and ‘’suicide behavior’’. Additionally, reference lists of the relevant case-control studies, review, and meta-analysis were reviewed manually to identify additional relevant studies.

### Inclusion and Exclusion Criteria

Studies included in our meta-analysis had to meet all of the following criteria: 1) used case-control study or cohort study; 2) the studies evaluated the associations between the COMT Val158Met (158G/A) polymorphism and suicide risk; 3) studies published in English and Persian; and provided sufficient information for calculation of odds ratio (ORs) with 95% confidence interval (CI). Accordingly, the following exclusion criteria were also considered: 1) those studies that were not designed as case-control or cohort studies; 2) control population including suicide attempter or other similar conditions; 3) there was no available genotype or allele frequency; 4) duplicate of previous publication; and 5) studies not written in English or Persian. Moreover, if multiple studies from the same case series were available, the one including the more individuals was used in the analysis.

### Data Extraction

Information was carefully extracted from all eligible publications independently by two investigators according to the inclusion criteria listed above and data with discrepancies in identification were discussed by all authors. For each included study, the following information was collected: first author’s name, year of publication, country of origin, total number of cases and controls, distribution of genotypes and alleles, minor allele frequencies (MAFs), and *P*-value for Hardy–Weinberg equilibrium (HWE). The countries of origin were categorized as Asian, Caucasian, and African.

### Statistical Analysis

The associations between the COMT Val158Met (158G/A) polymorphism and suicide were measured by odds ratios (OR) with 95% confidence intervals (CI) under the allele model (A vs. G), the homozygote model (AA vs. GG), the heterozygote model (AG vs. GG), the dominant model (AA+AG vs. GG), and the recessive model (AA vs. AG +GG). The heterogeneity assumption was checked by the Q-statistics with *P*-values < 0.1, and I^2^ statistics was calculated to quantify the proportion of the total variation across studies due to heterogeneity^14^. I^2^ values of 25%, 50%, and 75% were nominally assigned as low, moderate, and high estimates, respectively. When heterogeneity was considered significant, the random-effects model (DerSimonian- Laird approach) was performed. Otherwise, the fixed-effects model (Mantel-Haenszel approach) was used^15^. The departure from the Hardy–Weinberg equilibrium (HWE) for the control group in each study was tested using an online HWE calculator. The HWE was considered statistically significant when the *P*-value was less than 0.05. Sensitivity analysis was also tested by removing one study at a time to calculate the overall homogeneity and effect size. Publication bias was evaluated by funnel plots and further assessed by Egger’s linear regression test^16, 17^. All the statistical analyses were performed by comprehensive meta-analysis (CMA) ver. 2.0 (Chicago, IL, USA) software (Biostat, USA). All the *P* values were 2-sided. *P-*value less than 0.05 was considered statistically significant.

## Results

### Characteristics of Studies

Thirty-seven publications fulfilled our search criteria were preliminarily were enrolled. After reading the titles and abstracts, 22 studies with duplicate titles, review, meta-analyses or focused on unrelated condition were excluded. One study was excluded because was not case–control. Finally, 14 case-control studies^8, 9, 18-29^, on COMT genotypes and suicide risk were identified, including a total of 2353 suicide attempters and 2593 controls from 2000 to 2016 (Table 1). All studies were published in English. Among them five studies were in North America (USA, Canada, and Mexico), five in Asia (Taipei, Japan, and North Korea), and six (Germany, Croatia, Slovenia, Finnish Switzerland and France) in Europe. The genotypes in the healthy control group for two studies were not consistent with HWE (*P*<0.05)^23, 29^. Table 2 summarizes the difference of genotypes and alleles frequency between males and females suicide attempters.

**Table 1:** Main characteristics of studies included in this meta-analysis

**First author**	**Country (Ethnicity)**	**Genotyping** **technique**	**Case/** **Control**	**Cases**					**Controls**					**MAFs** ^a^	**HWE** ^b^
				**Genotypes**			**Allele**		**Genotypes**			**Allele**			
				**GG**	**AG**	**AA**	**G**	**A**	**GG**	**AG**	**AA**	**G**	**A**		
Ohara et al 1998 ^18^	Japan (Asian)	RFLP-PCR	12/135	2	7	3	11	13	58	59	18	175	95	0.351	0.627
Nolan et al 2000 ^8^	USA & Finnish (Caucasian)	No available	84/64	28	35	21	91	77	10	31	23	51	77	0.601	0.933
Russ et al 2000 ^19^	USA (Caucasian)	RFLP-PCR	49/49	9	28	12	46	52	7	26	16	40	58	0.591	0.491
Liou et al 2001 ^20^	Taipei (Asian)	RFLP-PCR	62/188	36	23	3	95	29	98	79	11	275	101	0.268	0.341
Rujescu et al 2003 ^21^	Germany (Caucasian)	RFLP-PCR	149/328	35	69	45	139	159	78	167	83	323	333	0.507	0.737
Ono et al 2004 ^22^	Japan (Asian)	RFLP-PCR	163/169	68	79	16	215	111	90	61	18	241	97	0.287	0.125
Baud et al 2007 ^23^	Switzerland & France(Caucasian)	RFLP-PCR	427/185	124	218	85	466	388	34	107	44	175	195	0.527	0.029
Zalsman et al 2008 ^24^	USA (Caucasian)	RFLP-PCR	200/119	34	114	53	106	294	27	67	25	121	117	0.491	0.168
Lee et al 2011 ^25^	Korea (Asian)	RFLP-PCR	197/170	94	85	18	273	121	69	85	16	223	117	0.344	0.160
Pivac et al 2011 ^26^	Slovenia (Caucasian)	Sequencing	356/198	78	197	81	353	359	45	97	56	187	209	0.527	0.809
Nedic et al 2011 ^27^	Croatia (Caucasian)	TaqMan	82/311	9	38	35	56	108	76	170	65	322	300	0.482	0.095
TovillaZárate et al 2011 ^9^	México (Mixed)	TaqMan	105/236	34	58	13	126	84	80	112	44	272	200	0.423	0.664
Du et al 2014 ^28^	Canada (Caucasian)	RFLP-PCR	98/72	16	19	14	51	47	22	30	20	74	70	0.486	0.158
Sun et al. 2016 ^29^	China (Asian)	LCR^c^	369/369	218	129	22	565	173	193	161	15	547	191	0.258	0.008

^a^Minor allele frequencies, ^b^Hardy–Weinberg equilibrium, ^c^Ligase chain reaction (LCR).

**Table 2:** Results of meta-analysis for the COMT Val158Met (158G/A) polymorphism and suicide risk

**First author**	**Country (Ethnicity)**	**Case/** **Control**	**Cases**					**MAFs** ^a^	**Controls**					**MAFs** ^a^	**HWE** ^b^
			**Genotypes**			**Allele**			**Genotypes**			**Allele**			
			**GG**	**AG**	**AA**	**G**	**A**		**GG**	**AG**	**AA**	**G**	**A**		
**Male Attempters**														
Ono et al 2004 ^22^	Japan (Asian)	112/114	43	60	9	146	78	0.348	62	42	10	166	62	0.271	0.457
Lee et al 2011 ^25^	Korea (Asian)	70/85	37	26	7	100	40	0.285	26	48	11	100	70	0.411	0.126
Pivac et al 2011 ^26^	Slovenia(Caucasian)	269/132	60	156	53	276	262	0.487	25	64	43	114	150	0.568	0.891
Nedic et al2011 ^27^	Croatia (Caucasian)	59/253	6	27	26	39	79	0.669	62	141	50	265	241	0.476	0.062
Du et al 2014 ^28^	Canada (Caucasian)	35/47	13	16	6	42	28	0.400	15	18	14	48	46	0.489	0.109
Sun et al.2016 ^29^	China (Asian)	117/117	70	40	7	180	54	0.230	64	46	7	174	60	0.256	0.737
Total		662/745	229	325	108	783	541	0.408	254	356	135	864	626	0.420	0.598
**Female Attempters**														
Ono et al 2004 ^22^	Japan (Asian)	51/55	25	19	7	69	33	0.323	28	19	8	75	35	0.318	0.130
Lee et al 2011 ^25^	Korea (Asian)	127/85	57	59	11	173	81	0.318	43	37	5	123	47	0.276	0.416
Pivac et al 2011 ^26^	Slovenia(Caucasian)	87/66	18	41	28	77	97	0.557	20	33	13	73	59	0.447	0.926
Nedic et al 2011 ^27^	Croatia (Caucasian)	23/58	3	11	9	17	29	0.630	14	29	15	57	59	0.508	0.998
Du et al 2014 ^28^	Canada (Caucasian)	14/24	3	3	8	9	19	0.678	7	11	6	25	23	0.479	0.688
Sun et al. 2016 ^29^	China (Asian)	252/252	148	89	15	385	119	0.236	129	115	8	373	131	0.259	0.003
Total		554/540	254	222	78	730	378	0.341	241	244	55	726	354	0.327	0.555

^a^Minor allele frequencies, ^b^Hardy–Weinberg equilibrium

### Pooled Analysis

Table 3 listed the main results of the meta-analysis of COMT Val158Met (158G/A) polymorphism and suicide risk. When all the eligible studies were pooled into the meta-analysis of COMT Val158Met (158G/A) polymorphism, there was no significantly increased risk of suicide under all genetic models (A vs. G: OR=1.089; 95% CI: 0.797, 1.488, *P*=0.592; AA vs. GG: OR=1.012; 95% CI: 0.726, 1.411, *P*= 0.943; AG vs. GG: OR=0.958; 95% CI: 0.762, 1.204, *P*= 0.714; AA+AG vs. GG: OR=0.889; 95% CI: 0.669, 1.182, *P*=0.419; and, AA vs. AG +GG: OR=0.980, 95% CI: 0.746, 1.286, *P*= 0.883).

**Table 3:** Results of genetic comparisons for COMT 158 G/A (COMT Val158Met) polymorphism and suicide.

**Genetic model**	**No. of studies**	**Model**	**Heterogeneity**		**Odds Ratio**		**Publication Bias**	
			**I** ^ 2 ^ ** (%)**	***P*** ** value**	**OR (95% CI)**	***P*** ** value**	**P**_Beggs_	**P**_Eggers_
**Overall**								
A vs. G	14	Random	91.00	0.001	1.089 (0.797, 1.488)	0.592	0.273	0.696
AA vs. GG	14	Random	61.17	0.001	1.012 (0.726, 1.411)	0.943	0.381	0.558
AG vs. GG	14	Random	55.72	0.006	0.958 (0.762, 1.204)	0.714	0.443	0.315
AA+AG vs. GG	14	Random	74.78	0.001	0.889 (0.669, 1.182)	0.419	0.458	0.722
AA vs. AG +GG	14	Random	61.05	0.001	0.980 (0.746, 1.286)	0.883	0.742	0.784
**Caucasian**								
A vs. G	8	Random	92.04	0.001	0.962 (0.631, 1.467)	0.858	0.901	0.750
AA vs. GG	8	Random	75.49	0.001	1.014 (0.611, 1.682)	0.958	0.901	0.877
AG vs. GG	8	Random	53.24	0.036	0.916 (0.680, 1.234)	0.566	0.901	0.993
AA+AG vs. GG	8	Random	83.63	0.001	0.829 (0.492, 1.397)	0.481	0.901	0.913
AA vs. AG +GG	8	Random	75.56	0.001	0.957 (0.650, 1.409)	0.824	0.901	0.573
**Asian**								
A vs. G	5	Fixed	51.18	0.085	0.966 (0.830, 1.124)	0.652	0.086	0.294
AA vs. GG	5	Fixed	0.00	0.424	1.051 (0.712, 1.550)	0.803	0.086	0.239
AG vs. GG	5	Fixed	0.00	0.450	0.871 (0.592, 1.282)	0.483	0.806	0.890
AA+AG vs. GG	5	Random	92.78	0.001	0.749 (0.311, 1.799)	0.518	0.806	0.966
AA vs. AG +GG	5	Fixed	0.00	0.687	1.135 (0.784, 1.644)	0.502	1.000	0.763
**Mixed**								
A vs. G	1	Fixed	0.00	1.000	0.907 (0.651, 1.262)	0.562	NA^a^	NA^a^
AA vs. GG	1	Fixed	0.00	1.000	1.438 (0.688, 3.007)	0.334	NA^a^	NA^a^
AG vs. GG	1	Fixed	0.00	1.000	1.753 (0.875, 3.513)	0.114	NA^a^	NA^a^
AA+AG vs. GG	1	Fixed	0.00	1.000	1.071 (0.656, 1.747)	0.784	NA^a^	NA^a^
AA vs. AG +GG	1	Fixed	0.00	1.000	0.617 (0.317, 1.201)	0.155	NA^a^	NA^a^
**By HWE** ^b^								
A vs. G	12	Random	91.57	0.001	1.150 (0.787, 1.681)	0.470	0.450	0.872
AA vs. GG	12	Random	59.34	0.005	1.065 (0.736, 1.541)	0.738	0.731	0.886
AG vs. GG	12	Random	40.93	0.068	1.067 (0.844, 1.348)	0.588	0.837	0.934
AA+AG vs. GG	12	Random	75.22	0.001	0.953 (0.678, 1.340)	0.781	0.945	0.899
AA vs. AG +GG	12	Random	64.30	0.001	0.964 (0.700, 1.328)	0.824	0.537	0.568

^a^Not applicable, ^b^ Hardy–Weinberg equilibrium

### Subgroup Analysis

Stratified analysis by ethnicity showed no association between COMT Val158Met (158G/A) polymorphism and suicide risk in the Caucasian, Asian and mixed populations. The same results were also revealed by HWE status (Table 3). Results of the stratified analysis studies for female and male attempters are summarized in Table 3. In the stratified analysis for male attempters, the data on genotypes of the COMT Val158Met (158G/A) polymorphism was available in six studies including 662 cases and 745 controls. However, the COMT Val158Met (158G/A) polymorphism was not significantly associated with suicide risk under all genetic models. For female attempters, the data on genotypes of the COMT Val158Met polymorphism was available in six studies including 554 cases and 540 controls. The COMT Val158Met (158G/A) polymorphism was associated with an increased the risk of suicide under the homozygote model (AA vs. GG: OR=1.829; 95% CI: 1.158, 2.889, *P*=0.010) and the recessive model (AA vs. AG +GG: OR=1.787; 95% CI: 1.195, 2.671, *P*=0.005) in females (Table 4). Additionally, there was no association between COMT Val158Met (158G/A) polymorphism and suicide risk by genotyping technique (Table 4).

**Table 4:** Results of genetic comparisons for COMT 158 G/A (COMT Val158Met) polymorphism and suicide.

**Genetic Model**	**No. of studies**	**Model**	**Heterogeneity**		**Odds ratio**		**Publication Bias**	
			**I** ^ 2 ^ ** (%)**	***P*** ** value**	**OR (95% CI)**	***P*** ** value**	**P**_Beggs_	**P**_Eggers_
**By Gender**								
**Male**								
A vs. G	6	Random	82.42	0.001	0.972 (0.644, 1.468)	0.894	1.000	0.931
AA vs. GG	6	Random	74.37	0.002	0.950 (0.439, 2.059)	0.898	1.000	0.624
AG vs. GG	6	Random	70.08	0.005	1.028 (0.621, 1.702)	0.913	0.707	0.993
AA+AG vs. GG	6	Random	75.36	0.001	1.000 (0.591, 1.692)	0.999	0.707	0.839
AA vs. AG +GG	6	Random	79.31	0.001	0.923 (0.451, 1.889)	0.827	1.000	0.987
**Female**								
A vs. G	6	Fixed	39.29	0.144	1.137 (0.943, 1.370)	0.180	0.259	0.050
AA vs. GG	6	Fixed	0.00	0.816	1.829 (1.158, 2.889)	0.010	0.707	0.540
AG vs. GG	6	Fixed	13.92	0.325	0.897 (0.690, 1.167)	0.418	1.000	0.218
AA+AG vs. GG	6	Fixed	27.38	0.229	0.996 (0.775, 1.280)	0.977	0.707	0.053
AA vs. AG +GG	6	Fixed	0.00	0.735	1.787 (1.195, 2.671)	0.005	0.707	0.791
**By Genotyping Technique**								
**RFLP-PCR**								
A vs. G	9	Random	93.20	0.001	1.169 (0.715, 1.911)	0.534	0.348	0.842
AA vs. GG	9	Fixed	34.53	0.142	0.954 (0.739, 1.231)	0.716	0.916	0.460
AG vs. GG	9	Random	54.08	0.026	0.959 (0.709, 1.296)	0.784	0.465	0.438
AA+AG vs. GG	9	Random	78.01	0.001	0.851 (0.566, 1.279)	0.437	0.916	0.876
AA vs. AG +GG	9	Fixed	21.66	0.250	0.962 (0.780, 1.187)	0.720	0.602	0.740
**TaqMan **								
A vs. G	2	Random	90.89	0.001	1.366 (0.608, 3.067)	0.450	NA^a^	NA^a^
AA vs. GG	2	Random	91.21	0.001	1.765 (0.280, 11.120)	0.545	NA^a^	NA^a^
AG vs. GG	2	Fixed	0.00	0.356	1.391 (0.908, 2.132)	0.129	NA^a^	NA^a^
AA+AG vs. GG	2	Random	74.50	0.048	1.603 (0.669, 3.840)	0.290	NA^a^	NA^a^
AA vs. AG +GG	2	Fixed	91.98	0.001	1.339 (0.302, 5.933)	0.701	NA^a^	NA^a^

^a^Not applicable

### Sensitivity Analysis

To evaluate the robustness of the association results, the meta-analysis was performed repeatedly with each study removed. No individual study significantly affected the pooled ORs. Sensitivity analysis was performed based on HWE, and the corresponding pooled ORs were not materially altered, indicating that our results were statistically robust.

### Publication bias

**Figure 1:**
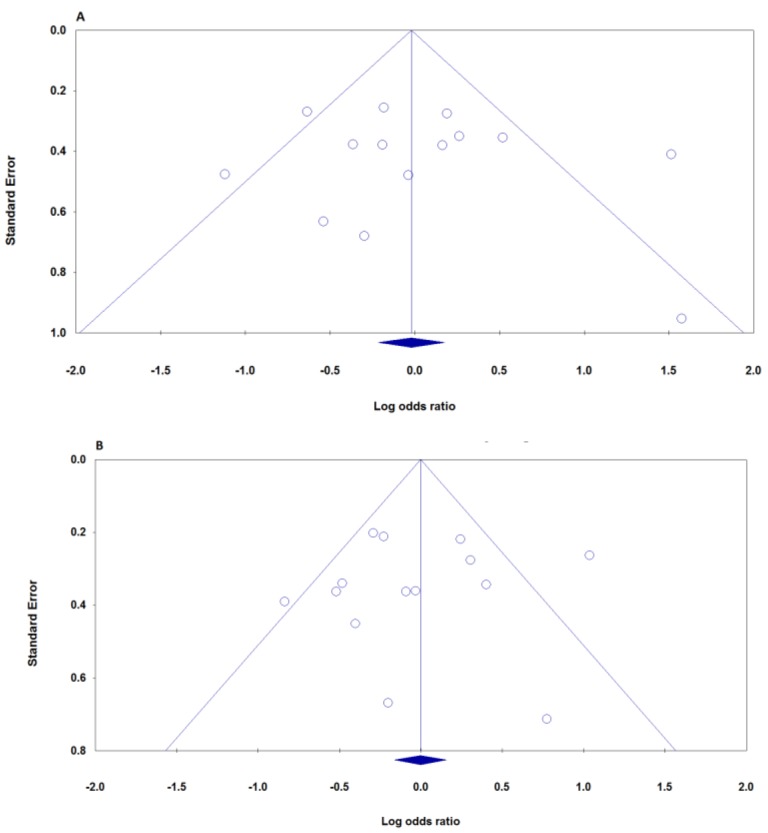
Begg’s funnel plots of the COMT 158 G/A (COMT Val158Met) polymorphism and suicide risk for publication bias test. **A:** the homozygote model (AA vs. GG) and **B:** the recessive model (AA vs. AG +GG).

Publication bias was qualitatively examined using the funnel plots and quantitatively estimated by Begg’s test and Egger’s test. For all genetic models, the shapes of the funnel plots did not reveal any evidence of obvious asymmetry (Figure 1). These results were further supported by analysis via Egger’s linear regression test (A vs. G: P_Beggs_= 0.273, P_Eggers_= 0.696; AA vs. GG: P_Beggs_= 0.381, P_Eggers_= 0.558; AG vs. GG: P_Beggs_= 0.443, P_Eggers_= 0.315; AA+AG vs. GG: P_Beggs_= 0.458, P_Eggers_= 0.722; and AA vs. AG +GG: P_Beggs_= 0.742, P_Eggers_= 0.784, Table 1).

## Discussion

To date, the association between COMT Val158Met polymorphism and suicide remains controversial because the reports include both positive and negative findings^9, 30^. Several large studies in the USA, Germany, Slovenia, and Croatia suicide attempter cases found no evidence for an increased risk of suicide^21, 24, 26^. These epidemiological studies results are inconsistent because this association was found in presence of the COMT Met allele in the male and female suicide attempters and diagnosed with different mental and mood disorders such as schizophrenia and alcohol dependency.. These differences among studies might be explained by the different diagnostic entities used. Therefore, the main limitation could be that the genetic association studies on COMT 158G/A polymorphism have been performed in different ethnicity and may be different criteria have been applied in the studies to define suicidal behavior, which reduces power to find consistent results^21, 24, 31^.To the best knowledge, the present meta-analysis is the most comprehensive and accurate meta-analysis assessed the association between the COMT 158G/A (COMT Val158Met) polymorphism and suicide risk.‏ Our results did not show any‏ significant association between the COMT Val158Met polymorphism and suicide in overall. Several issues should be further discussed to address the negative results. However, for most 14 studies involved, when they were individually removed from the analysis, the association between COMT Val158Met polymorphism and suicidal behavior was not significant still. Thus, the evidence for an association between COMT and suicidal behavior was robust.

Our results were consistent with a more recent meta-analysis on COMT Val158Met and suicide risk. Eleven case–control studies and one case-only study were included on COMT Val158Met polymorphism^9^. COMT Val158Met polymorphism was not associated with suicidal behavior risk. However, COMT Val158Met and suicide risk essentially remain an open field, as meta-analysis study not selected healthy controls (with only 875 adult suicide attempters). Therefore, their results’ reliability and the number of studies are considerably smaller than that needed to receive the robust conclusions^32^. Moreover, compared with their meta-analysis, subgroup analysis by ethnicity and gender were also carried out. In our meta-analysis, the COMT Val158Met polymorphism was associated with suicide risk in females.

Interestingly, when all the eligible studies were pooled into male and gender subgroups analysis, only in female subgroup increased risk for suicide was found. However, there was an imbalance in gender distribution, with more males in attempters than females. None of the genetic models showed association with male susceptibility, suggesting that COMT 158G/A (COMT Val158Met) manifest female specific influences on suicide attempt. Estrogen in females modulates neurotransmission and neuronal excitability of catecholaminergic systems and increases the concentration of dopamine in the synaptic gap, leading to the different influence of dopamine on emotion and motivation among male and females ^33,,34^. In addition, gender-related differences in suicide behavior might be due to the fact that COMT 158G/A (COMT Val158Met) polymorphism may differently influence personality traits that presumably affect suicide behavior^35, 36^. However, we were unable to conduct accurate more analyses of COMT polymorphisms by gender subgroup for insufficient data, thus the existence of gender-specific influences on suicide in this meta-analysis remains unclear. In this meta-analysis the female specificity in effect of the COMT 158G/A (COMT Val158Met) polymorphism on suicide attempt could be a chance finding, this result is inconsistent with gender differences in the dopamine system^37^. Further examination in the included eligible case-control studies in the gender subgroups showed significant differences between cases and controls numbers in male and female subgroups.

In interpreting results of this meta-analysis, some limitations should be acknowledged. First, not all the eligible studies were collected; the sample size of the included studies was large enough by ethnicity, especially in non-Caucasian and Asian populations. Therefore, there was a lack of statistical power to evaluate better the association between COMT 158G/A (COMT Val158Met) polymorphism and suicide, especially in subgroup analysis. Second, due to limited individual data, a more precise analysis on other covariates such as age, number of suicide attempts, and environmental factors were not performed. Third, since the genotypes frequency of COMT 158G/A polymorphism not mentioned by gender of suicide attempters in all studies, we only evaluated the eligible studies for gender subgroups. Finally, as suicide is a complex trait, gene-gene and gene-environment interactions were not analyzed. Specific environmental and lifestyle factors will alter the associations between gene polymorphisms and suicide susceptibility. Therefore, it is necessary to evaluate the roles of lifestyles in suicide.

## Conclusions

The present meta-analysis is the most comprehensive and accurate meta-analysis assessed the association between the COMT 158G/A (COMT Val158Met) polymorphism and suicide risk. COMT Val158Met polymorphism was not associated with suicide risk in overall population. However, subgroup analysis by gender showed that COMT Val158Met polymorphism was strongly associated with suicide risk in the female subgroup. More comprehensive studies and larger samples are necessary to determine conclusively an association of COMT 158G/A (COMT Val158Met) with suicidal behavior.

## Acknowledgment

The authors would like to thank Professor Seyed Mahdi Kalantar and Hasan Sheikhha for impetus to perform this study.

## Conflict of interest statement

We have no conflict of interest to declare

## Funding

No grants were involved in supporting this work.

## Highlights

- The COMT Val158Met polymorphism is not significantly associated with suicide risk in overall analysis.
- Stratified analysis showed no association between COMT Val158Met polymorphism and suicide by ethnicity.
- The COMT Val158Met polymorphism does appear to be of major importance in the susceptibility of suicide in females
